# Fluctuations and remaining bonds: Challenging undynamic fetal personhood through women's experiences of early pregnancy endings in England

**DOI:** 10.1111/maq.70061

**Published:** 2026-03-09

**Authors:** Susie Kilshaw

**Affiliations:** ^1^ Department of Anthropology University College London London UK

**Keywords:** miscarriage, personhood, pregnancy, pregnancy endings

## Abstract

Women's subjective relationship with their pregnancy is central in understanding fetal personhood, a relationship that is theirs to assemble and disassemble. A rigid perception of personhood as either present or absent is problematized, instead revealing an evolving approach. Based on twenty months of ethnographic fieldwork in an NHS Trust in England the paper explores women's experiences of personhood during pregnancy, pregnancy endings, and their aftermath. It shows personhood is neither fixed, nor constant, but rather oscillates; revealing that dichotomous understandings of personhood based on a specific moment, and which are unidirectional, are flawed. Women's experiences add further evidence that personhood is not biological and is not always tied to the fetus or fetal body. Instead, it is social relations including an imagined future that confers personhood with these sometimes continuing after the pregnancy ending. Yet, social relations that bring a fetus into being may also unmake it.

## INTRODUCTION

When personhood begins and ends is central to anthropological theorizing (Kaufman & Morgan 2005; Layne 2022). Anthropologists have shown that personhood is not just a biological fact, but a social and cultural achievement; contested, relational, and deeply meaningful. In the first anthropological work on pregnancy loss, Roseanne Cecil (1996, 1) recognized the importance of “the stage (pre‐ or post‐partum) at which the fetus/baby is attributed with human status” and its cultural and historical variation. Since then, social scientists have consistently demonstrated the contingent nature of fetal and infant personhood, including key contributions to this journal. Scholars have revealed how fetal personhood is negotiated through various practices, such as the use of material goods by women in the US (Layne 2000, 2003) and acts like naming and imagining a future (Middlemiss 2024). The distinction between a fetus and a baby is not by gestational development but social relationships (Layne 2003; Middlemiss 2024) which continue to evolve with women as agents in this process.

In this article, based on ethnographic fieldwork, I develop the concept of fluctuating personhood in England, as it shifts throughout a pregnancy, the duration of a miscarriage, and continues to evolve beyond. Informed by Middlemiss (2024; also Fuller, Littlemore, & McGuinness 2025) the paper considers the way that experience of time and materiality may extend beyond the ending of the pregnancy and miscarriage, contributing to an understanding that transcends the concept of personhood. The paper contributes to scholarly work exploring the ontologies of the not‐alive fetal body (Fuller & Kuberska 2020; Middlemiss 2021, 2024) whilst also problematizing notions of the fetal body itself. As a medical anthropologist specializing in miscarriage, my research contributes to a growing body of work that aims to transform the way we discuss fetuses and produce ways to acknowledge them as subjects without ceding reproductive options, rights, and agency for women (Oaks 2000, 67). Claims about the ontological status of pregnancy materials^1^ are not value‐free and have significant implications for a variety of practices. A key consequence of definitions of personhood is in relation to abortion. The meanings ascribed to fetuses and the history of efforts to grant social identities to them is a problematic topic in feminist thought with the primary concern being that to focus on the fetus surrenders the anti‐abortion movement its major premise and forecloses the feminist insistence on reproductive freedom for women (Michaels & Morgan 1999, 1). However, the notion of fetal personhood only threatens feminist reproductive politics if one assumes a narrow model of “inherent” and biologically determined personhood (Layne 2003; Morgan 1996) that anthropological scholarship has consistently undermined. With such an approach, it is clear that the process of constructing personhood may be undertaken with some embryos and not others (Layne 1997), allowing for the recognition of fetal personhood without negating abortion access. Furthermore, it provides space for acknowledging loss and/or grief in pregnancy endings without inevitably granting inherent personhood.

### Anthropological approaches to personhood

Personhood has been shown to be co‐developed by clinicians and their use of biomedical technology as they materialize fetuses through sound mediated by fetal Dopplers (Howes‐Mischel 2016) and through imaging. Ultrasound technology has been shown to be central to the construction of fetal personhood in a variety of ethnographic contexts (Ivry 2010; Mitchell 2001). My recent publications (Kilshaw 2024a, b) contribute to the body of work that demonstrates how clinical practices produce fetal personhood (Howes‐Mischel 2016; Mitchell 2016) in England (Kuberska 2020; Middlemiss 2021, 2024). Scholars have problematized dichotomous systems of personhood based on a specific moment revealing nuanced navigations in conferring personhood in medical settings (Christoffersen‐Deb 2012; Weiner 2009). Manon Lefevre (2025) moves beyond formations of “personhood” to scrutinize how IVF embryos are enacted through varied practices not only as unborn children but as many kinds of objects, revealing their “ontological capaciousness.” Astrid Christoffersen‐Deb (2012) shows how the viable fetus is a culturally specific product of the US political and legal imagination that “brings to life” conditional persons that blur distinctions between born and not‐yet‐born. Others have also destabilized the link between personhood and birth showing that personhood construction continues after birth in some contexts (Weiner 2009; Scheper‐Hughes 1993). Aimee Middlemiss (2024) challenges the idea that personhood is solely defined by biological criteria such as viability or birth and argues it emerges through relationships, acts of care, and emotional investment. This relational process confers personhood even when the law or medicine does not, with women resisting systems that deny personhood of their babies.

Along with others I problematize systems of personhood based on a specific moment but do so by emphasizing the way that personhood construction may travel in multiple directions. Whereas my previous work focused on the production of personhood by clinical disposal practices and its navigations by women, this paper focuses on the complexity of personhood with a focus on flux. It engages with rich anthropological literature, highlighting personhood as non‐linear, negotiated, reversible, conditional (Christoffersen‐Deb 2012; Weiner 2009), and messy (Verdaguer 2019), contributing nuanced, oscillating lived experience of personhood. Discussion of personhood as continuously assembled, assigned, contested and withdrawn within shifting regimes of power, affect, and embodiment (Chen 2012; Wolf‐Meyer 2025, 2020) is helpful. The way some (i.e. radicalized, disabled and queered) bodies are sometimes rendered less‐than‐person, overly animated, or treated as matter out of place (Chen 2012) is productive in thinking about how fetuses/ pregnancy tissue may be overly animated by some and de‐animated by others. The way in which “persons” emerge through institutional practices including bureaucratic classifications, medicine, and legal systems and how institutions actively fabricate personhood (Wolf‐Meyer 2020, 2025) resonates with the infrastructural relations that give rise to or deny fetal personhood.

Marilyn Strathern (1992) argues that persons in the English context are imagined as separate individuals located in bodies, yet in practice are made through relations with death not necessarily a barrier to continued personhood. Alfred Gell (1998) departs from the English individualism Strathern describes revealing how material things actively extend, mediate, and embody personhood. Personhood is not confined to the individual body but distributed across social relationship and material forms. This work is fertile ground to explore fetal personhood, for the fetus is simultaneously part of the woman and constituted through social, biomedical, and kinship relations. Miscarriage exposes relational complexity. Pregnancy tissue is neither fully individuated nor simply part of the maternal body; instead, it occupies an ambiguous space where decisions about naming, caring for, or discarding become moments where kinship, embodiment, and personhood are actively negotiated or denied. In pregnancy endings, personhood is negotiated, suspended, or reconfigured through social relations, clinical practices, and material indexes such as ultrasounds and the handling of remains.

Anthropologists Lynn Morgan and Meredith Michaels argue that ideas about the fetus cannot be relegated to anti‐abortion advocates. Linda Layne called for anthropologists and others to move beyond the paralysis that has prevented articulating feminist agendas with the issue of pregnancy loss close to thirty years ago (1997). This paper contributes to this agenda by adding further evidence of variable personhood formation, but also its construction and deconstruction beyond the end of the pregnancy. Furthermore, such shifts may include the material fetus or move away from it. Social relations that bring a fetus into being may also unmake it, and personhood in pregnancy defies focus on the fetus/ fetal body. The paper goes beyond the notion that a fetus is either granted personhood or not but instead reveals how attributions are not static: they wax and wane, are conceived and constructed in an ongoing way. There may be parallel ontologies. Relevant to this is the disentanglement of the biological and notions of personhood and temporal consideration beyond the pregnancy ending. I argue that personhood as an object is subjective and shifting, lending support for a feminist rethinking of the system that would allow women greater agency to define how they understand their pregnancy (Middlemiss 2024) and the beings or non‐beings it might contain.

This research sits in a wider framework of a feminist approach to pregnancy endings. Scholars have highlighted ambiguity in pregnancy calling for a need to problematize assumptions around pregnancy outcomes. Sara DiCaglio (2017) suggests that the future defines embryos’ present, whereas there are many possible outcomes, the majority of which are not the production of a child. Victoria Browne (2022) has called for disentangling pregnancy from birth and rethinking what pregnancy can amount to besides the production of a baby, suggesting an alternate philosophy of pregnancy that welcomes variation, requiring us to sit with uncertainty and ambiguity. In Browne and Kilshaw (2026) we have argued for an inclusive full‐spectrum approach to pregnancy endings. Along with McGuinness and others, we emphasize that miscarriage and abortion “sit on a spectrum of experiences that intersect and overlap experientially and medically as well as legally” (McGuinness 2025), and as such need to be approached connectedly. We argue that if bodily autonomy is forwarded as the core principle a sense of loss can be validated just as much as a sense of relief or happiness because what matters is whether people are empowered to determine the meaning of their own pregnancy ending. In line with this, this paper contributes evidence of the complex experience of pregnancy endings and personhood. The women I have spent time with over the past 14 years experience nuances of personhood, sometimes reflecting on how their understandings relate to their thoughts about the boundaries of life, their feelings toward living children and dead loved ones, notions of motherhood as well as abortion. Women express nuanced, complex and shifting experiences of personhood which may be fused to the materiality of the fetus and/or the imagined child, may shift between the two, or be held as separate, as will be discussed below. Personhood also relates to the elapsed time of the pregnancy, its ending, the process of the miscarriage, and time beyond the experience. Women's experience of time and materiality might extend the experience of pregnancy beyond its ending.

## METHODS

The paper is based on data from ethnographic research including participant observation and semi‐structured interviews with women experiencing miscarriage (n37) and those involved in their care (n38) over a twenty‐month period between February 2021 and September 2023.^2^ The research design was informed by previous research (Kilshaw 2020) and explored the remains and residues of miscarriage broadly defined. Women were recruited at one NHS Trust in England. For observation sessions participating staff would provide women with the study information and seek verbal consent. If both agreed, I would be invited into the room. I observed all elements of miscarriage care including diagnosis, ultrasound scanning, miscarriage management, and consenting for management and remains disposal. Disposal pathways and memorialization activities were observed. Observations were not recorded, and no personal information was collected. Some participants took part in both observations and interviews.

Participation for the interview component was offered on posters and flyers displayed in the site and by clinic staff familiar with the study. Twenty‐seven women self‐referred, and after expressing interest via email or phone call were again sent the information sheet and consent form and given an opportunity to ask questions. I sought and recorded informed consent to participate prior to the interview. I interviewed women during or soon after their miscarriage and follow‐up interviews were conducted three to six months later. No participants withdrew consent. I interviewed ten women who participated in my previous research and were treated in the same NHS Trust to explore long‐term remains of miscarriage, including ongoing acts of memorialization or their absence, and to probe their experience of remains disposal. Participants volunteered a variety of reproductive histories, and many had experienced earlier pregnancy endings including miscarriages, ectopic pregnancies, abortions, and one stillbirth. These experiences were often used by participants as points of resonance or comparison, and several participants had had experiences of both home and clinically managed miscarriage.

I conducted most interviews in person, but due to COVID‐19 restrictions a small number were conducted online. Individual, semi‐structured interviews lasted forty‐five to ninety minutes as “guided conversations” (Lofland & Lofland 1984) with respondents encouraged to give their own accounts and meanings in relation to the main research questions; their experience of the remains of miscarriage broadly defined, and their experience of disposal. I recorded field notes after each interview. Audio recordings were professionally transcribed verbatim. Field notes and interview transcripts were analyzed using a thematic analysis approach; the method was iterative and based on a grounded theory approach (Strauss & Corbin 1990). Whilst the research included miscarriages up to twenty‐three weeks and six days, all but one participant experienced first trimester miscarriage, and this is the focus of the paper.

I conducted interviews with thirty‐eight health professionals (i.e. doctors, nurses, sonographers, chaplains) involved in the care of miscarriage or handling of pregnancy tissue. In addition to formal interviews, informal exchanges and ongoing conversations during fieldwork provided additional data. Whilst a small number of health professional participants disclosed previous experiences of miscarriage, or other forms of reproductive experiences, none became a miscarriage participant. Participants were pseudonymized. The research was granted full ethics approval by the NHS HRA Ethics Committee.

This research is grounded in a feminist anthropological framework that recognizes knowledge as situated, embodied, and relational. The ethnography foregrounds lived experiences, making visible the silences, inequalities, and affective dimensions of pregnancy endings that are often erased. In this sense, ethnography offers not only description but an ethical and political intervention into how pregnancy endings are understood, valued, and addressed. Acutely aware of my role in meeting women in intimate and vulnerable settings and during a sensitive time; I approached interviews as dialogic encounters rather than one‐sided extractions of information. A key element of this was that all participants self‐referred, were given time to consider participation. As a researcher who has experienced three miscarriages, my positionality is not peripheral to this project: it is central to the questions I ask, the relationships I build, and the ways I interpret and present narratives shared. My experience informs both my intellectual and emotional investment in this work and I would refer to it when appropriate. While I do not assume that my experience mirrors those of the individuals I spoke with, it shaped my sensitivity to the diversity and ambiguities that often surround pregnancy endings. That two of my miscarriages were treated in the same NHS Trust but over fifteen years previously provided institutional knowledge and an awareness of how miscarriage discourse and care have changed. Ultimately, the research seeks not only to document experiences, ensure a diversity of voices, and improve clinical care, but contribute to a broader feminist effort to acknowledge the fetus as a subject whilst also advocating for women's reproductive agency.

## FLUCTUATIONS

Fetuses are rapidly turned into children and women into mothers, yet these transformations can be reversed, or roles denied (Verdaguer 2019; Middlemiss 2021, 2024). In the US when a pregnancy is lost so is the woman's status as a mother (Layne 2003). Alice Lovell (1983) showed how attitudes and procedures around pregnancy loss deconstruct the identity of a mother and a baby in cases of perinatal loss in England whilst Maria Verdaguer (2019) describes the making and unmaking of both mother and baby during her experience of ectopic pregnancy. Being a mother is entwined with the personhood of the pregnancy: it is the child that makes a mother. A woman is a mother in relation to a child either as someone who has given birth or who fulfills a nurturing and caregiving role in a child's life. Motherhood is dependent on the presence of the other being, something which some women grappled with in the face of miscarriage. Personhood may be processual and related to duration of pregnancy and/or stage of fetal development (Middlemiss & Kilshaw 2023) and shifts as one moves through the miscarriage experience (Kilshaw 2024c). Personhood is not a static analytic category but a fluid and volitional social practice; it is repeatedly recreated by people entwined in multiple social contexts (Morgan 1996, 63). People may imbue personhood to a pregnancy, embryo, or fetus and then renegotiate, shear, or remove it at different stages of the pregnancy, the pregnancy ending, or its aftermath. Some women resist constructions of personhood, such as those found in clinical practices, challenging the medicalization of temporality and materiality.

### Experiencing fluctuating motherhood

Some women I met saw themselves as mothers, as Jennifer did: she became a mother when she became pregnant, and did not relinquish this role when her pregnancy ended:

You didn't just lose a pregnancy, you lost the one‐year‐old you imagined and the five‐year‐old on the first day of school and the grown up that you pictured in your mind, even in just those few short weeks… Even though they might not have been public and it might not have been a child you met and held… It was your child… “I'm a mum too.” Just because I'm not pushing a buggy or going to the school gate, it doesn't mean that I wasn't a mum.

Jennifer states, “I'm a mum too” and later suggests her motherhood was real, but in the past, suggesting shifting motherhood and ambiguity. For Jennifer, motherhood is linked not only to her lost pregnancy but the imagined child and extends beyond her pregnancy ending. Other women express uncertainty and ambivalence around motherhood: questioning whether they continue to be a mother after their pregnancy ending or, indeed, whether they had ever been a mother. Ambiguity lies in uncertainty as to whether there is a person to whom one's motherhood is in relation.

For some the sense of a miscarriage being the loss of a baby diminishes if they have living children. This may be because they focus on the enhanced personhood of a child compared to that of a fetus or related to motherhood, as Nina explains:

I suppose we felt …it wasn't a baby, it wasn't a child. We didn't feel that there was a need for a more formal ritual around a goodbye… a burial or cremation… With all of these pregnancy losses I haven't lost being a mother, I am a mother. And I think that's a huge protective factor. I wonder if I would need the losses to have more meaning if I didn't already have that. Maybe I didn't need the pregnancy to have the meaning of the loss of a child because I had a child and it felt really different.

For Nina her understanding of her pregnancy ending and whether she considered it as the loss of a person relates to motherhood conferred through her son.

### Abrupt conclusions

During her treatment for ectopic pregnancy, Verdaguer was given drugs that caused the demise of the embryo, leading her to wonder when the pregnancy stopped and asking whether it was once she knew the embryo wouldn't become a baby (2019, 32). Women I have interviewed similarly express ambiguity as to when their pregnancy ended and when there ceased to be a (potential) baby. Women described the moment when they are informed that they have miscarried as “the moment of loss.” Katie explains diagnosis was followed by a process of reframing the pregnancy and “distancing” herself: “by the time” she “gets to the point of” surgical management,

You're in a very different head space and you just carry on moving beyond that in terms of dissociating from the event.

Hence discussions about disposal which accompanied the preparation for surgery were at odds with the way she understood her pregnancy at this stage:

I saw another couple who had come out being given the leaflet… I think the moment of leaving with the leaflet^3^ is the worst moment, isn't it? It's when everything falls apart. There are worse moments after that, but it is the defining moment… but [the discussion around disposal] did surprise me because I was in a completely different frame of mind, and I think by that point you've also done a lot of the hard work to stop yourself mentally being attached to the baby that would have been. So, you've worked really hard to think of it not as a baby and then you're forced to think of it as a baby.

Katie describes an active process of deconstructing personhood or navigating away from it, which is challenged by hospital practices that frame the miscarriage as the loss of a baby (REOVED). Women who undergo surgical management of their miscarriage typically have had days if not weeks pass between the diagnosis and surgery, with many describing a transformative process where the pregnancy is no longer understood as containing a baby but rather biological matter. Katie describes “working hard” to stop being attached to “the baby that would have been” suggesting potential personhood, or personhood in relation to the imagined baby rather than the being itself. There is ontological fluidity which Katie actively negotiates. She moves between thinking of her pregnancy as containing a “baby” to something “that would have been,” suggesting it is not (yet) something to be attached to. Personhood is messy rather than linear; making and unmaking a child may occur concurrently (Verdaguer 2019, 34).

Medical practices emphasize the moment or process of expulsion of the material as central to miscarriage experiences, which is unsurprising given the centrality of birth for understandings of pregnancy (Browne 2022), but for Katie and many others it is the diagnosis that is central. Like Katie, Laura suggests the bond with the fetal being ends abruptly at the point of diagnosis:

It's weird because when I was pregnant, I was attached to the baby or fetus or whatever. I very much wanted that pregnancy, but once I know it's going to be over, I don't really get sentimental about the remains. It sounds bad, but I really wouldn't want a pot of ashes.

Laura was uninterested in engaging in discussions about disposal and found the suggestion of funeral director involvement and cremation inappropriate although her experience was of bereavement. Laura's experience highlights nuanced elements of attachment and personhood. Attachment points to a social and intimate relationship and relational selves, which are key in shaping fetal personhood (Middlemiss 2024). Having had several early scans, Katie's experience of developing relations was informed by technology, one scan revealing:

A heartbeat. Weirdly enough there was a second sac, which was empty. I theoretically already sort of lost one, but I didn't really count it as a loss. I felt like we didn't lose what we didn't have… there's no point dwelling on it. We've still got one, we've got a chance. Then I had another scan, maybe two weeks after that, and it was fine, and it was growing well.

Laura's experience reveals the flexible and subtle nature of personhood. Laura did not feel a sense of loss for the twin pregnancy because it had been unknown to her. Her focus was on the continuing pregnancy: the fetus with a beating heart. Yet the attachment to the fetal being ends with diagnosis; when she undergoes surgical management and the accompanying discussion of disposal, she has a lack of sentimentality about the biological material.

## Pregnancy as Illness and Risk

Some women experience their ongoing miscarriage as illness, as Meredith did:

For a while now it hasn't been a pregnancy for me, it's been an illness, a chronic illness that I've been dealing with and trying to get better from.

For some women the embryo, fetus and/or pregnancy material becomes something dangerous, as experienced by Verdaguer (2019, 32) who describes a process whereby her embryo became an illness. Referring to Boltanski's distinction (2004) between the “project” fetus and the “tumor” fetus, Verdaguer describes a process of understanding her embryo as less and less human and more as a cancer and parasite, documenting the shift from her focus on the embryo as child to herself as she came to understand that her life was in danger.^4^ Similarly, Nina's relationship with her pregnancy changed as the danger her ectopic pregnancy posed dawned:

Because my own life, without being dramatic, I think I read something on one of the websites, “The leading cause of death in pregnancy in the UK is ectopic pregnancy,” … and I was in a lot of pain… I think that the shock of needing to have surgery… the idea that I was at threat and therefore my relationship with my son was at threat… felt like that was all at risk so, it changed how I felt about the material. It was posing a risk with the child I do have… in that moment I feel like I chose myself and my family that I do have, the reality of my life, not a fantasy about what could have been. And that has been quite anchoring.

For Nina, the reality of her life and her child is held in contrast to the “fantasy” of “what could have been” and project fetus. Instead, the biological material is not a baby, but pain, risk, and threat:

I think my relationship [with the pregnancy] changed… it was sort of a foreign body. My son described it [laughs]: “Was it like a germ, was it like a bad bit,” I was saying, “Well, it wasn't exactly like a germ…” but it was. You're sacrificing… it's not someone but something else to save yourself in that situation. I felt grateful after the change in law in America about abortion. I felt really grateful to live somewhere where my health and wellbeing was prioritized over that of the material.

Nina touches on how abortion practices are relevant to how women understand pregnancy material. Several women referred to abortion, and particularly abortion debates in the US, when they spoke about their experience. Nina's understanding shifted as she moves through the experience of diagnosis, treatment, pregnancy ending, and beyond. For some personhood recedes as treatment continues and the pregnancy material becomes something hazardous requiring removal. This process is linked to the separation between the materiality of the fetus and/or the pregnancy tissue and personhood, as will be explored in the next section.

### Fluctuating relationship between biological material and experiences of loss

For some women the biological material may be entangled with grief: there is a desire to retain, manage, and dispose of it in a ceremonial way; personhood may be connected to the materiality of the fetal body and/or pregnancy tissue. I witnessed women who had miscarried at home bury the remains or seek the help of a funeral director to have them cremated. Marianne kept the ashes of her two miscarried pregnancies in small wooden boxes in her bedroom. She expressed ongoing attachment to them; however, this was complicated by the fact that one likely did not contain any fetal remains. A participant in the previous research, Sophie had buried her baby in her garden. During an interview for the current research she told me the grave now had the addition of a small statue representing the baby. Memorialization had continued and remained active, but “softened.” This resonates with Fuller, Littlemore, and McGuinness's (2025) work that highlighted the depth of relationships that people feel toward their babies/fetal beings, bonds that may continue beyond death. Like one case they discuss, the ashes become metonymic for Marianne exceeding “a clinical and conventional British English understanding of ‘the remains.’” For some women the connection between materiality and the experience of grief extends to memorialization practices around the remains, such as having a grave or preserving the ashes.

I witnessed women being careful to retain tissue to take to the clinic and others who had miscarriages managed by the hospital relieved when they were informed of their choices for disposal. Women who understood their miscarriage to be the loss of a baby felt supported by clinical practices; yet for others their understanding of their pregnancy tissue contrasts with clinical practices. For some, upset and feelings of loss in early miscarriage are disentangled from the biological matter. Laura and Nina were both disinterested in what happened to the pregnancy tissue, which was common amongst the women I met. Whilst typically, but not always, upset about the ending of their pregnancy, women did not consider the material to be the fulcrum of loss (Kilshaw 2024a), as Meredith explains:

I think they were quite separate. And I think the loss is more of a concept for me than a physical thing.

Meredith's actions in the months and years after her miscarriage reflect her understanding of loss as a concept, as discussed below, but also how personhood is complex and evolving. Alex also did not have an “emotional attachment” to her pregnancy tissue:

That's not to say I wasn't upset, there was a lot of crying and upset about what could have been but not really upset about [what it] was at that point.

Whilst sad, for Alex it was not a “baby loss” but of “what could have been.” Like many women, Grace flushed her pregnancy tissue unceremoniously, an act which did not reflect a lack of upset. I first met Grace when the consultant called me into the room during her follow‐up appointment, explaining that Grace, also a doctor, was struggling to make sense of her feelings of grief. She later described differing approaches to her pregnancies based on her fertility journey:

With my first baby we couldn't get pregnant and we had treatment to have her. And that was sort of four years of trying and so when I became pregnant with her, I couldn't think of her as a baby. She was hope but until she was in my arms, I couldn't believe that she would happen… in some respects this one was more of a baby because I was already planning… I was cautious… but in my head… this could happen. There could be a baby. So, you need to think about how you're going to manage this and that and so… baby feels like the wrong term but all the impersonal sort of ball of cells, like whilst that's comforting in a miscarriage setting, that doesn't feel right either.

For Grace, personhood is based on reproductive intentions, time and effort spent conceiving, pregnancy duration, but also planning a future. In trying to understand why she felt grief she noted “another layer of significance.” She and her husband had decided they would no longer try to conceive after her fortieth birthday, but she had conceived after this date.

I'm really struggling with that and it doesn't feel justified, part of me is attaching a lot of that to this pregnancy. But I do have this sort of sense of wanting to do something, but not really knowing what and feeling foolish that I would want to do something but also sort of knowing it would just be for me.

When I met with Grace some months later, she showed me the small charm she had bought and attached to a necklace she always wore. The charm rested at the nape of her neck, and she explained it was something for only her to remember the loss. For Grace and Alex, the pregnancy material is not connected to feelings of loss and yet clinical practices around disposal assume their entanglement (Kilshaw 2024a and c). Scarlett describes her pregnancy as containing “a clump of cells with hopes and dreams attached;” her pregnancy ending is not the loss of a baby, but she imagines the child that could have been, and is upset about plans unmade, and the loss of a particular future. Of all the women I met, Scarlett was the most frustrated and distressed with clinical practices that framed her miscarriage as the loss of a baby. Many women resisted the medicalization of the materiality of their pregnancy ending. In attempting to provide sensitive care, clinical practices may unwittingly be insensitive to some and be providing resonance with understandings found amongst anti‐abortion advocates who locate death in biological material rather than social personhood (see Cromer & Bjork‐James 2023).

Some women engage in memorialization activities entirely separate from the materiality of the pregnancy: buying jewelry or obtaining a baby loss certificate. Notions of personhood may not be tied to the biological fetal being. This understanding is different from seeing the pregnancy as a corpse/body that is the focus of loss and that should be appropriately disposed of and mourned. It is for this reason that I argue for destabilizing clinical practices that assume miscarriage as bereavement (Kilshaw 2024a) and emphasize biological materiality. Instead, I call for the creation of flexible systems that accommodate diverse responses to pregnancy endings and their materials. Such an approach provides space for parallel ontologies and shifting notions of personhood over the course of a pregnancy ending and beyond.

### Presence or absence of fetal body

Above I have provided evidence of experience of loss as disentangled from pregnancy tissue and described the way in which personhood is relational rather than fixed on biological criteria (Middlemiss 2024). Relational and disorderly understandings of personhood provide space for those who may mourn a pregnancy not containing an embryo, such as those who test positive for pregnancy but are not actually pregnant—in the case of an empty embryonic sac or a molar pregnancy (Layne 2003). Such understandings accommodate experiences such as those I witnessed at the clinic of women who had started bleeding very soon after a very early positive pregnancy test who in previous contexts would have been experiencing a normal or slightly delayed menstrual period. Some of these women experienced loss and bereavement. Berend's (2010, 2016) research revealed that for surrogates in the US, chemical pregnancies are considered miscarriages despite the lack of presence of an embryo because of the focus on the imagined child, with distinctions between egg, embryo, fetus, and baby are often erased. For some women the lack of an embryo or fetus does not diminish feelings of personhood, but for others it may lead to its absence, as it did for Nicole:

There was no baby to grieve. It would be different if it were a fetus. I had already grieved and knew there wasn't a baby there. So, for me it was just get it [tissue] out. I want this to be over and start afresh.

Nicole explains that her sense of grief is over the “idea of a baby” and describes a desire to rid her body of the tissue. Here we see a lack of personhood, with Nicole describing relief once the material exited her body and “flinging” it in the toilet and flushing. My second miscarriage was an anembryonic pregnancy, which I responded to with more ambivalence than my previous miscarriage and later miscarriage, which both contained fetuses. Whilst the lack of an embryo informed my lesser feelings of upset, this was merely part of a configuration of factors including the shorter pregnancy duration, the lack of materiality of personhood through ultrasound imagery and detection of fetal heartbeat, my ambivalence with the pregnancy itself, and that this was the only miscarriage that was signaled by bleeding prior to diagnosis.

Hannah was one of the few women I interviewed who collected (some) of her pregnancy tissue when she miscarried at home. Her desire to collect the remains surprised her as did her decision to retain them, by burying them in a pot under a house plant. She explained:

It's biological matter but for those few weeks it wasn't.

Hannah had miscarried on the toilet, as many do. She let most of the material slip through but collected the “last clot” which she understood to be the embryo. Hannah later reflected on her experience in trying to make sense of it. She described the miscarriage of her five‐week embryo conceived through IVF as one of a “long line of disappointments” and that the miscarriage symbolized the “end of the road” for her and her partner conceiving their biological baby. She knew that this IVF round would be her last with her own egg.

Maisie, like Hannah, buried her pregnancy remains. However, her experience differed in several ways. Maisie passed some blood and tissue at home, but most of her pregnancy emerged in clinical settings. She understands her baby to have been removed in the hospital when she was rushed there in a critical state. A few weeks later she came to the EPAU to undergo surgery to remove “retained tissue,” which is when I met her. Soon after arriving at the clinic, the clinical team prepared her for surgery,^5^ obtaining informed consent for the procedure and discussing disposal. When asked what she would like done with the tissue Maisie waved her hand and said, “do what you like, I'm not interested.” She explained the “baby” had already been removed during her previous hospital visit; “anything” removed during this surgical procedure was “not baby related.”

During an interview a few weeks later, Maisie spoke about her feelings toward her pregnancy:

I just felt this attachment to this thing that honestly looked no more than a peanut on the screen… that was my child.

Referring to her five‐week embryo as her “child” she treated the remains as a body requiring burial and memorialization. Maisie points out that she and her partner approached her pregnancy differently: Mark did not share her notions of personhood. For Mark the tissue was risky and damaging to the woman he loved. Describing himself as “scientific,” he did not think of it as a baby. Mark was surprised that Maisie brought the remains home and was shocked that this was something offered by the hospital. Yet he was supportive of Maisie's choice to bury them in a garden pot. She named the baby and explained that she wanted the remains to stay close to her.

These examples show variation in notions of personhood and how they are not innate, with those with a relationship to the pregnancy, pregnancy material, or baby having different understandings of its personhood. In the case of Maisie and Hannah, we see ascriptions of personhood as linked to the biological entity with more import placed on what they understand to be the fetus or embryo. It also reveals ambiguity, for whilst Maisie was sure that the fetus was removed in the clinic, Hannah thinks the “last clot” she collected was the “pregnancy” but isn't certain. Many women I met spoke about uncertainty and ambiguity in terms of the biological material of pregnancy endings. This was often exacerbated in cases, which were common, when the pregnancy tissue was expelled over a duration and in different locations. Revealing prenatal and posthumous personhoods exist and are not either/or but on a continuum containing different forms of meaning built on different experiences of the world and the body, Middlemiss (2024) shows personhood may be linked to materiality of the individual body yet argues that in the case of pregnancy endings personhood is fluid.

### Fluctuating and remaining bonds

Above I discussed how Meredith and others came to understand their miscarriage as an illness, and how clinical practices that assumed bereavement were challenging to the meaning of their pregnancy tissue. Experiences of personhood shift as women travel through the experience. This process may continue beyond their pregnancy ending and the duration of their miscarriage, as illustrated by Meredith. Meredith and I stayed in touch after she participated in the research, and I observed how her experience of personhood evolved months after her pregnancy ending. Eager to advocate for those experiencing miscarriage, she wrote a blog for her workplace. When she sent it to me, I was struck by her reference to her miscarriage as a bereavement, something which seemed to contradict the way she had framed her experience previously. She explained that her emphasis on bereavement was, in part, due to her wanting her audience to understand the gravity of the experience. Yet it was more than this, as reflected by her reporting to me that she had applied for a baby loss certificate: I began to see how the personhood of her first pregnancy had continued to shift some time after its ending:

With the first miscarriage I was working through as more of a medical problem. It was in part the only way to cope with how difficult and drawn out it was, but it was also in part a conscious choice linked to my personal approach to life. I don't know if my position would have stayed the same if we'd have stopped our pregnancy journey there, but life goes on and I have really noticed my perspective continually evolving ever since.

This materialized in a change in the language Meredith used to refer to her experience, which was impacted by her subsequent miscarriage followed by an ongoing pregnancy. The experience of being pregnant following miscarriage was central to Meredith's understanding of her experience:

I've pivoted from “miscarriage” to “pregnancy loss” because it relates now to my experience of “pregnancy after loss,” and I see—and want to retain—a connection between the two.

Meredith explained that she had shifted to framing her experience as bereavement, outlining several influencing factors: the duration of her “fertility journey” and the lack of care from friends and family meant the “losses” became more “loaded and significant.” As she awaited the birth of her daughter, she reflected that the past four years had been dominated by pregnancy and pregnancy endings with the losses “becoming more important” as a result.

A major factor in the way she perceived the “fetuses” was her experience of her ongoing and “successful” pregnancy.

I had chosen not to view my two first pregnancies as babies, however, getting into my second trimester and feeling the daily movement gave me a real sense of the baby as independent being, and this really changed how I felt about the first two—they were independent beings too, I just couldn't feel it yet.

After her second miscarriage and whilst pregnant Meredith took on a role which meant she spoke about her experience regularly to others, which meant she reflected on her experiences of miscarriage and “pregnancy after loss.” This meant her miscarriage experiences were “kept in the present,” informing an emphasis on the losses and their personhood. Part of this was her desire to help others who had experienced miscarriage, which “naturally leads to placing greater significance on my own losses.” Meredith's understanding of the personhood of her fetal being shifted months after her pregnancy ending and miscarriage, informed by her current situation and experiences.

## CONCLUSIONS

Women's experiences highlight the complexity of ascriptions of personhood, calling for the need to pay heed to nuance, diversity, and contingency. I have shown how a model of innate, fixed, biological, and constant personhood is challenged by women's experiences. Instead, personhood is subjective and shifting. I have shown how social relations that bring babies into being may also unmake them and that personhood in pregnancy is not always entangled with the fetal body. In the clinic there is a focus on the biological material as central to the experience of miscarriage and/or loss. The focus in clinic settings is on materiality, but also the temporal nature of the pregnancy and miscarriage with certain practices linked to gestation. Pregnancy and miscarriage in the clinic are conceptualized in terms of objective time, which is resisted by some women whose experience of subjective time is emphasized. The paper has described women's resistance to the medicalization of materiality and temporality. The women discussed in this paper refer to heartbeats and scans as informing personhood, but diagnosis can disrupt personhood either abruptly or gradually. Once the potential for further development of the pregnancy and personhood is no more, some women stop ascribing personhood to the fetal being. In some cases, personhood based on the material fetus shifts to a personhood located with the imagined child. In this way, the paper contributes to key anthropological discussions about personhood and when a being becomes a social person.

Within an increasingly restrictive reproductive landscape, medical anthropologists and scholars of reproduction must work to decipher the many different realities embryos (Lefevre 2025), fetuses, fetal beings, and pregnancy tissue occupy. My research lends weight to Middlemiss's (2024, 229) call to “establish a system which responds to women's clinical and emotional needs rather than judges the gravity of their experience on the basis of the fetal body and its prospective outcomes,” which requires an ontological shift placing women's needs alongside those of the fetal being in all pregnancies, not just those producing a living person. This is part of a wider call by feminist scholars to challenge the view of pregnancy as a means to an end, rather than a meaningful embodied experience regardless of how it ends (Middlemiss 2024, 229–30; see also Browne 2022). I wholeheartedly agree that what is needed is less prescription and assumptions of grief in clinical care, and instead flexible systems more inclusive of the diversity of experiences, including shifting and dynamic understandings of personhood. Linked to this is a need to move away from “restrictive ontological positions and [to] acknowledge and respect diversity in ontologies of the person” (Middlemiss 2024, 230) in all forms of pregnancy endings. Women should be able to define their pregnancies, pregnancy tissue, and/or fetal beings in a way that feels comfortable to them and should be supported in doing so.

## CONFLICT OF INTEREST STATEMENT

The author declares they do not have competing interests. The research was supported by the Wellcome Trust as part of a University Award in the Social and Historical Science (Award number: 212731/Z/18/Z)

## ETHICAL APPROVAL

The research had full ethics approval from NHS HRA Ethics Committee (Integrated Research Application System (IRAS) Reference: 261330, Research and Development Reference: PID14448‐SI001, Research Ethics Committee Reference: 19/SC/0428The research was supported by the Wellcome Trust as part of a University Award in the Social and Historical Science (Award number: 212731/Z/18/Z)Note: if the paper is accepted for publication I would like a truncated ethics statement, if possible in order to protect the site of the research and maintain anonymity

## ENDNOTES

1 I use the term “pregnancy material” as an inclusive term to refer to pregnancy tissue, blood, clots, as well as embryonic or fetal tissue. The term also is meant to point to the ambiguity of what is contained in pregnancy and pregnancy endings, particularly early pregnancy. The term is also meant as a neutral term to avoid connotations of meaning with “pregnancy remains”.

2 The research was suspended between June 2021 and May 2022 due to the COVID‐19 pandemic.

3 The “leaflet” in question is the patient information sheet given to women after a miscarriage diagnosis, which outlines management options and other information.

4 Women I have interviewed similarly note a shift in attention from the embryo/fetus/pregnancy to themselves and their health during the process of a pregnancy ending. Women may shift, as Verdaguer does, from a (potential) mother to a patient.

5 In this case, manual vacuum aspiration, a surgical procedure used in miscarriage and termination of pregnancy where the pregnancy tissue is gently removed from the womb under a local anesthetic.
